# Impact of ultraviolet-B radiation on early-season morpho-physiological traits of *indica* and *japonica* rice genotypes

**DOI:** 10.3389/fpls.2024.1369397

**Published:** 2024-03-01

**Authors:** Sonal Mathur, Raju Bheemanahalli, Salah Hameed Jumaa, Naqeebullah Kakar, Vangimalla R. Reddy, Wei Gao, Kambham Raja Reddy

**Affiliations:** ^1^ Adaptive Cropping Systems Laboratory, United States Department of Agriculture-Agricultural Research Service (USDA-ARS), Beltsville, MD, United States; ^2^ Department of Plant and Soil Sciences, Mississippi State University, Mississippi State, Starkville, MS, United States; ^3^ United States Department of Agriculture (USDA) UVB Monitoring and Research Program, Department of Ecosystem Science and Sustainability, Colorado State University, Fort Collins, CO, United States

**Keywords:** combined UV response index, genetic variability, individual UV response index, root, shoot

## Abstract

Ultraviolet (UV)-B radiation is considered one of the major detrimental rays coming from the Sun. UV-B radiation has a harmful impact on plant growth and development. The effect of UV-B radiation was studied on 64 rice (*Oryza sativa* L.) genotypes during the vegetative season. An equal number of genotypes from the *japonica* (50%) and *indica* (50%) subspecies were phenotyped using the Soil-Plant-Atmosphere-Research (SPAR) units. The 10 kJ UV-B was imposed 12 days after planting (DAP) and continued for three weeks (21 d). Based on the combined ultraviolet-B radiation response index (CUVBRI) for each genotype, the 64 rice genotypes were classified into sensitive, moderately sensitive, moderately tolerant, and tolerant. Various shoot traits, such as plant height, tiller, and leaf numbers, were measured. We also studied critical root phenological traits like root volume, diameter, tips, and forks. Out of all the studied shoot traits, leaf area showed maximum reduction for both *indica* (54%) and *japonica* (48%). Among the root traits, root length decreased by negligible (1%) for *indica* as compared to *japonica* (5%), while root crossing and forks showed a maximum decline for *japonica* (37 and 42%), respectively. This study is timely, meaningful, and required because it will help breeders select a tolerant or sensitive rice line for better yield and production under abiotic stresses.

## Introduction

Sun rays are the principal source of energy received by the Earth. Out of all the radiation reaching the planet Earth, nearly 7-9% of the radiation is ultraviolet (UV) radiation (100-400 nm) (Frohnmeyer and Staiger, 2003). However, the ratio is mere and lethal for the entire living system. UV radiation is divided into three sub-radiations: UV-A (315-400 nm), UV-B (280-315 nm), and UV-C (100-280 nm). UV-C is the most hazardous of the three radiations to reach the Earth’s surface. Still, it is less critical for plant health because the atmosphere absorbs much of this radiation. The ozone layer absorbs approximately 95% of UV-B radiation and reaches the Earth’s surface with an average intensity of 1 Wm^−2^ (Cejka et al., 2011). Anthropogenic factors are a significant cause of the depletion of the ozone layer, leading to UV-B radiations on the Earth’s surface (UNEP, 2014). The amount of UV-B radiation has increased due to stratospheric ozone (O3) depletion caused by anthropogenic chlorofluorocarbons (Sah et al., 2022). In the future, changes in UV radiation reaching the Earth’s surface are expected to be influenced more by climate factors (cloud cover, aerosols, and surface reflectivity) than by changes in stratospheric ozone, assuming full compliance with the Montreal Protocol (Bernhard et al., 2020; Barnes et al., 2023; Bernhard et al., 2023). However, uncertainties persist. A recent study suggests a potential 3–8% increase in the UV Index over the tropics and mid-latitudes by 2100, depending on greenhouse gas scenarios, cloud cover, and aerosol concentrations. Additional factors affecting UV exposure include increased wildfires, which generate aerosols and harm the ozone layer, land-use practices (e.g., deforestation), melting of snow and ice, and shifts in species distribution due to climate change (Barnes et al., 2023). UV-B radiation is difficult to separate from the Sun’s radiation. Plants are sensitive to UV-B during crop growth and development (Kakani et al., 2003a). UV-B can affect plant’s morphology, anatomy, and physiology (Hollosy, 2002; Reddy et al., 2003; Kakani et al., 2003a; Kakani et al., 2003b; Mathur and Jajoo, 2015; Mathur et al., 2018; Ramamoorthy et al., 2022). Effects of UV-B have been studied in the field (Sullivan et al., 2007) and in horticulture crops (Craver et al., 2014). Studies on maize reported that UV-B radiations decrease the number of chloroplasts in the leaves (Santos et al., 1993; Fagerberg and Bornman, 1997), reduce rubisco activity, decrease stomata numbers and leaf structure (Lingakumar and Kulandaivelu, 1993). Likewise, studies in cotton crops showed adverse effects of UV-B on pigments, including leaf necrosis, chlorosis, and early leaf senescence (Reddy et al., 2003; Kakani et al., 2003b). Such inhibitory effects of UV-B led to a decrease in photosynthesis and protein biosynthesis in many crops such as cotton (Kakani et al., 2003a), rice (Kumagai et al., 2001), sugarcane (Yuan et al., 2011), buckwheat (Yao et al., 2008), corn (Singh et al., 2014; Wijewardana et al., 2016), wheat (Mathur and Jajoo, 2015; Mathur et al., 2018), sweet potato (Chen et al., 2020; Ramamoorthy et al., 2022).

Rice (*Oryza sativa* L.) is one of the most important crops, extensively cultivated around the globe. Irrigated rice accounts for approximately 55% of the global harvested area and contributes 75% of global rice production, with 25% use of global agricultural freshwater (Thanawong et al., 2014). Rice provides ~27% of dietary energy needs, ~20% of nutritional protein, and ~3% dietary fiber (Kennedy et al., 2003). Rice also contains bioactive compounds such as anthocyanins, flavonoids, and polyphenols (Fukagawa and Ziska, 2019). Therefore, rice cultivation has been under tremendous pressure to keep its productivity with population growth and demands.

UV-B can affect the morphological and physiological processes in rice. Teramura et al. (1991) reported that rice sensitivity is latitude-based. Asian rice genotypes have different sensitivity as compared to other rice genotypes. Rice exposed to UV-B at the vegetative stage negatively affects coleoptile growth (Idris et al., 2021), biomass-related traits, and yield-determining traits (Kumagai et al., 2001). In response to UV-B, rice leaves accumulate higher malondialdehyde (MDA) content (Du et al., 2011) and anthocyanin (in purple rice strain) (Hada et al., 2003). Rice genotypes were also screened for UV-B tolerance based on the various phenolic concentrations and biomass weight (Teramura et al., 1991; Dai et al., 1994; Cassi-Lit et al., 1997).

Few studies (Jumaa et al., 2020) focused on phenotyping rice genotypes for vegetative stages. A knowledge gap exists regarding the impact of UV-B stress on rice genotypes during the early vegetative stage, particularly concerning shoot and root physiology. Previous studies did not consider the phenotypic variability in shoot and root traits across various rice genotypes in response to UV-B stress during the vegetative stage. This study addresses this gap and enhances our understanding of the intricate interactions between UV-B stress and rice genotypes at the critical early vegetative stage. The specific objectives of the study were to (i) quantify UV-B induced variability in morpho-physiological at the early growth stage, (ii) determine the correlation between physiology and vigor under UV-B, and (iii) identify rice genotypes tolerant to UV-B stress at early vegetative stage. The study will be helpful in the future in identifying and selecting tolerant and susceptible rice lines or varieties for various abiotic stresses.

## Materials and methods

### Plant material and crop cultivation

A panel (64 genotypes) consisting of an equal number of genotypes from *japonica* (50%) and *indica* (50%) (**Supplementary Table 1**) were phenotyped using the sunlit controlled environment facility, known as Soil-Plant-Atmosphere-Research (SPAR) units at the Rodney Foil Plant Science Research Center, Mississippi State University, Mississippi State, MS, USA. Four uniform seeds of each genotype were sown in the 384 PVC plastic pots filled with gravel (300 g) at the bottom and a mixture of sand and topsoil with a 3:1 (v/v) ratio. Pots were arranged in a randomized complete block design (RCBD) with three replications, and each genotype was placed randomly. For irrigation, Hoagland’s nutrient solution (Hewitt, 1952) was provided three times based on 120% of the evapotranspiration measured in each SPAR unit as described in Reddy et al. (2001) using an automated and computer-controlled drip irrigation system with one dripper per pot.

### Treatment imposition

This experiment used 384 pots (64 rice genotypes x two treatments x three replications) to examine variability among rice genotypes for UV-B radiations. The ten kJ UV-B was imposed 12 days after planting (DAP) and continued for three weeks (21 d). A square-wave supplementation system (constant UV-B supplements) was used to provide desired UV-B radiation dosages, which were delivered from 0.5 m above the plant canopy for eight h each day, from 08:00 to 16:00 h by eight fluorescent UV-B-313 lamps (Q-Panel Company, Cleveland, OH, USA) mounted horizontally on a metal frame inside each SPAR chamber, driven by 40 W dimming ballasts. The UV-B radiation delivered at the top of the plant canopy was monitored at ten different locations in each SPAR chamber daily at 10:00 h with a UVX digital radiometer (UVP Inc., San Gabriel, CA, USA), which was calibrated against an Optronic Laboratory (Orlando FL, USA) Model 754 Spectroradiometer that was being used initially to quantify the lamp output. As needed, the lamp output was adjusted to maintain the desired UV-B level, as described by Reddy et al (2003, 2013). Across treatments, typical temperatures (30/22°C; day/night) were maintained during the experiments. Air temperature in each SPAR unit was monitored and adjusted every 10 s throughout the day and night. The mean temperature (day/night) and relative humidity (day) were 26.0 ± 0.8°C and 46.0 ± 13%, respectively. The (CO_2_) in each SPAR unit was monitored and adjusted every 10 s throughout the day and maintained within set points ± 10 µmol mol^-1^ as 420 µmol mol^–1^.

### Physiological trait measurement

#### Chlorophyll content, flavonoid and nitrogen balance index, chlorophyll fluorescence measurement

Physiological traits such as chlorophyll content, flavonoid, anthocyanin, and nitrogen balance index (NBI) were measured using a handheld Dualex Scientific instrument (Force A DX16641, Paris, France) at 33 DAP or 21 days after stress (DAS). The nitrogen balance index (NBI) was automatically calculated as a ratio of the chlorophyll content (ChI) to the flavonol index (FLAV), i.e., NBI = ChI/FLAV.

Chlorophyll fluorescence was measured using the FluorPen FP 100 (Photo System Instruments, Kolackova, Czech Republic). We followed the instrument protocol described by Reddy et al. (2021). To determine the maximum potential quantum efficiency of Photosystem II, fluorescence parameters such as maximal fluorescence intensity (Fm), maximal variable fluorescence (Fv), and the ratio of Fv to Fm (Fv/Fm) were measured (Reddy et al., 2021).

### Shoot and root trait measurements

Plant height, tiller numbers, and leaf number on the main axis were measured manually. Plant height was measured using the metric yard rule. The total leaf area was measured using the LI-3100 leaf-area meter (LI-COR, Inc., USA) 33 DAP or 21 DAS.

Individual plant’s roots and shoots were separated. The soil adhered to the roots and was washed thoroughly with a mild-speed water stream. Harvested roots were scanned using an Epson Expression 11000XL scanner at a resolution of 800 dots per inch, and the digital images were analyzed using WinRHIZO Pro 2009C software (Regent Instruments, Québec, Canada). Information extracted from the WinRHIZO software was used to determine the UV-B effect on root traits such as the longest root length, total root length, root surface area, root volume, average root diameter, root tips, root forks, and root crossings. Finally, we estimated the whole plant’s dry weight (leaf +stem +root) across treatments.

### Data analysis

Statistical analyses were carried out using RStudio 3.6.1 (https://rstudio.com/). Two-way ANOVA was performed on the phenotypic data to estimate the source of variation in genotype (G), treatment (T), and their interaction (G x T) using the library (“Agricolae”), in R, a language and environment (https://www.R-project.org/, R Core). Genotype or treatment means were compared with the Least Significant Difference (LSD) test, and the probability level of p< 0.05 was considered statistically significant. Sigma Plot 13.0 (Systat Software, Inc., San Jose, CA, USA) was used to plot all graphs.

Further, rice genotypes were classified into UV-B tolerant and sensitive groups based on the genotype response index (Reddy et al., 2021). Initially, the Individual UV-B response index (IUV-BRI) for each parameter was calculated as the value of a parameter (*P*l) at the 10 kJ of UV-B of a given genotype divided by the value for the same parameter (*P*o) at the optimum 0 kJ of UV-B (Equation 1).


(1) IUV−BRI=P1/Po

Then, each genotype’s Combined UV-B response index (CUV-BRI) (Equation 2) was calculated as the sum of all IUV-BRIs derived from plant height (PH), tiller number (TN), leaf number (LN), leaf area (LA), leaf dry weight (LWT), shoot dry weight (SWT), root dry weight (RWT), stem dry weight (StWT), root/shoot (RS), total dry mass (TDM), longest root length (LRL), total root length (TRL), root surface area (RSA), root diameter (RD), root volume (RV), Tips, Forks, crossings (Cr), quantum efficiency of Photosystem II (Fv/Fm), chlorophyll (Chl) content, flavonoid (Flav), anthocyanin (Anth), and nitrogen balance index (NBI).


(2) $$\begin{array}{l} \text{CUV}-\text{BRI}=(\text{PHl}/\text{PHo})+(\text{TNl}/\text{TNo})+(\text{LNl}/\text{LNo})+(\text{LAl}/\text{LAo})+\\ (\text{LWTl}/\text{LWTo})+(\text{SWTl}/\text{SWTo})+(\text{RWTl}/\text{RWTo})+(\text{StWTl}/\text{StWTo})+\\ (\text{RSl}/\text{RSo})+(\text{TDM}/\text{TDMo})+(\text{LRLl}/\text{LRLo})+(\text{TRLl}/\text{TRLo})+\\ (\text{RSAl}/\text{RSAo})+(\text{RDl}/\text{RDo})+(\text{RVl}/\text{RVo})+(\text{Tipsl}/\text{Tipso})+\\ (\text{Forksl}/\text{Forkso})+(\text{Crol}/\text{Croo})+(\text{Fv}/\text{Fml}/\text{Fv}/\text{Fmo})+(\text{Chl}1/\text{Chlo})+\\ \left(\text{Flavl}/\text{Flavo})+(\text{Anthl}/\text{Antho})+(\text{NBIl}/\text{NBIo}\right) \end{array}$$

## Results

### Phenotypic variability in response to UV-B

All the measured and derived phenotypic traits of 64 rice genotypes decreased (3 to 53%) in response to UV-B stress (**Supplementary Tables 2A, B**). Morphological characters such as plant height, tiller numbers, and leaves on the main axis varied between *indica* and *japonica* genotypes in response to UV-B.

**Figure 1:**
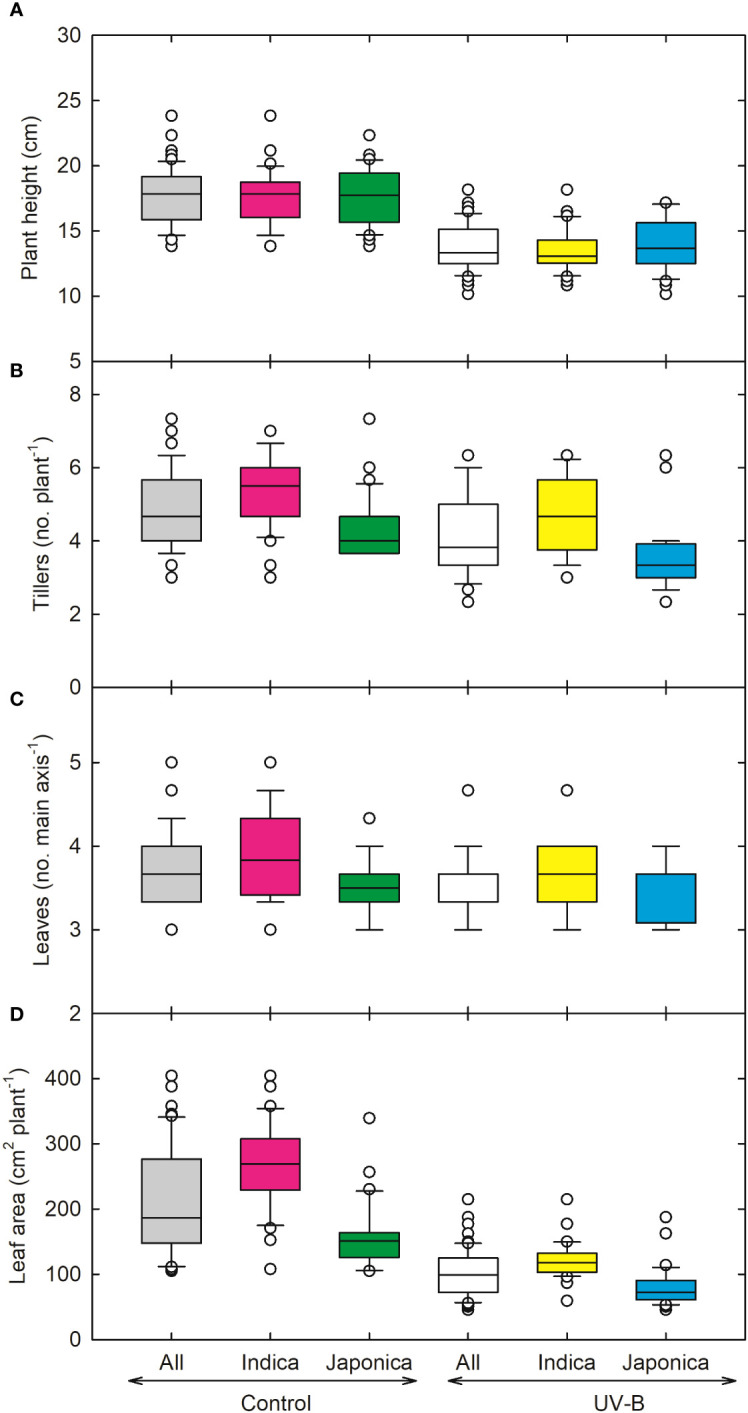
Ultraviolet-B radiation effects on shoot growth traits, **(A)** plant height, **(B)** tillers, **(C)** leaves on the main axis and **(D)** total leaf area of 64 *indica* and *japonica* rice genotypes. Measurements were taken 33 days after sowing and 21 days after UV-B treatment. The middle line indicates the median, and the box shows the range of the 25^th^ to 75^th^ percentiles of the total data. The whiskers indicate the interquartile range and the outer circle lines with high or low scores in each category.

**Figure 2:**
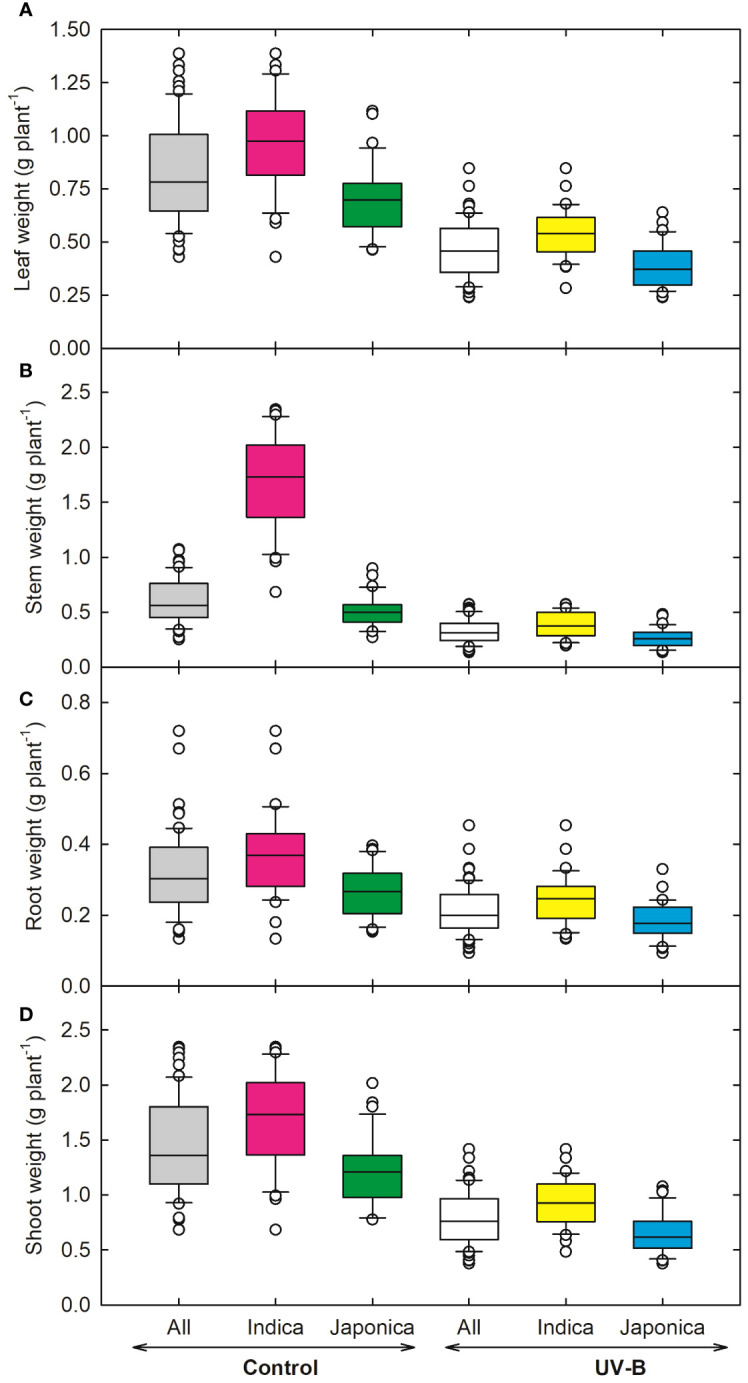
Ultraviolet-B radiation effects on shoot growth traits, **(A)** leaf weight, **(B)** stem weight, **(C)** root weight, and **(D)** shoot weight of 64 *indica* and *japonica* rice genotypes. Measurements were taken 33 days after sowing and 21 days after UV-B treatment. The middle line indicates the median, and the box shows the range of the 25^th^ to 75^th^ percentiles of the total data. The whiskers indicate the interquartile range, and the outer circle lines have high or low scores in each category.

In response to UV-B, plant height (PH) decreased by 24% in *indica* and 21% in *japonica* (**Figure 1A**). Genotype RU1504122 (*japonica*) showed the maximum decrease (10 cm) in plant height. In contrast, we recorded the highest PH (17 cm) for genotype RU1504114 (*japonica*). In *indica*, the tiller number (TN) was reduced by 10% and by 20% in *japonica* under UV-B (**Figure 1B**). Individually, the maximum decrease in TN was recorded for UV-B sensitive genotypes RU1204156 (*japonica*, 3 no. plant^-1^). Maximum values for TN were obtained for COLOMBIA XXI (*indica*, 5 no. plant^-1^). Under UV-B, leaf number on the main axis decreased to 7% and 2% for *indica and japonica*, respectively (**Figure 1C**). Maximum decrease in leaf number was observed for CT18247-12-8-1-4-2-2 (*indica*, 3 no. main axis^-1^), while the maximum increase in N-22 (*Aus*, 4 no. main axis^-1^) and NIPPONBARE (*japonica*, 4 no. main axis^-1^). Among all the 64 genotypes, RU1204156 (*japonica)* showed the lowest values for leaf area (57.2 cm^2^ plant^-1^) and recorded the highest value for MERMENTAU (*japonica*, 97 cm^2^ plant^-1^) (**Figure 1D**). As compared to the control, leaf weight, stem weight, root weight, and shoot weight were negatively affected under UV-B (**Figures 2A–D**). Compared to the control, the leaf weight of the *japonica* genotype (RU1204156) showed the least value (0.32 g plant^-1^), while RU1404156 recorded the maximum value. Stem weight ranged from 0.23 g plant^-1^ (*indica*, CT18244-9-4-4-2-1-2) to 0.47 g plant^-1^ (*japonica*, RU1404156). Likewise, for root weight, the *indica* genotype (CT18244-9-4-4-2-1-2) showed minimum values under UV-B.

**Figure 3:**
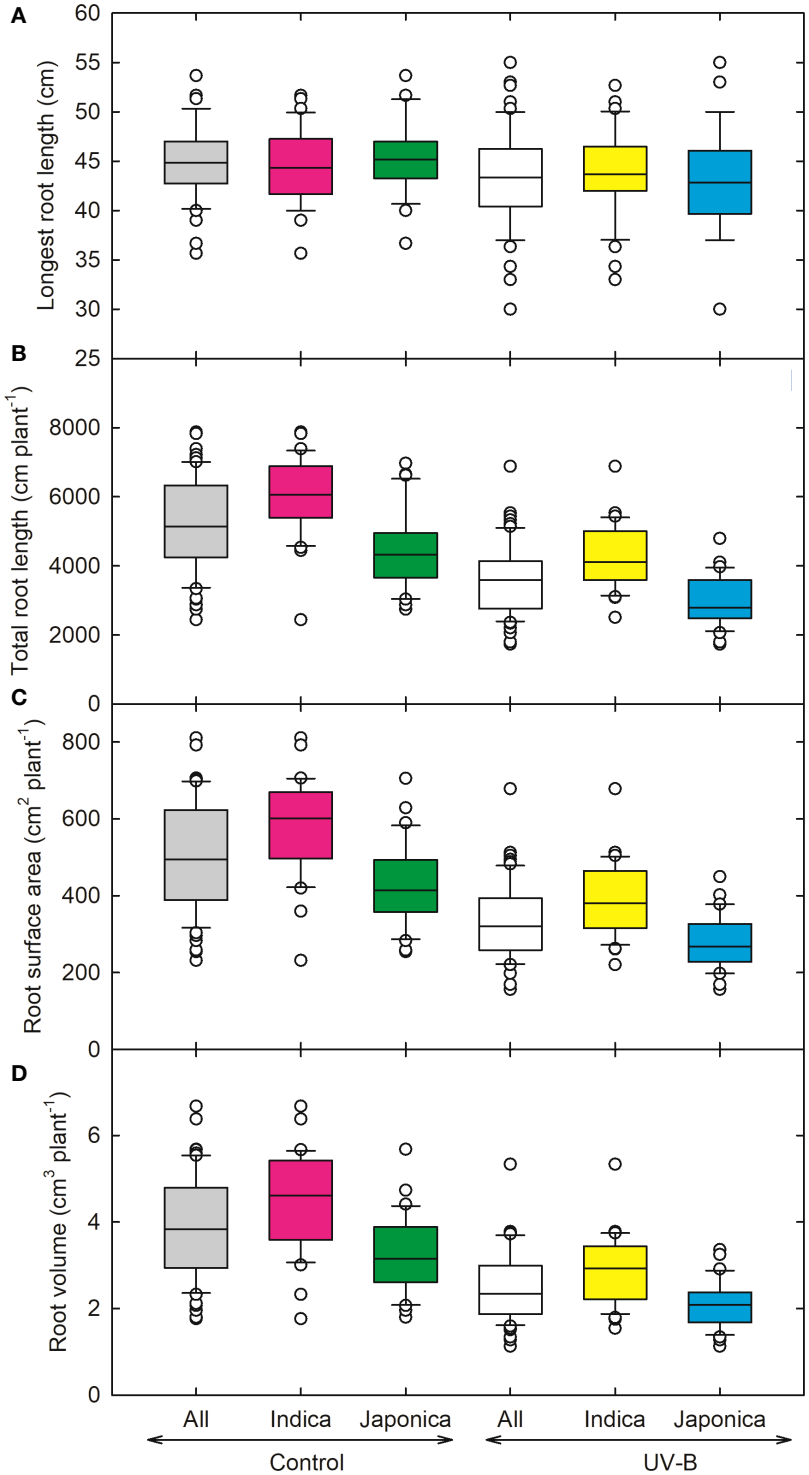
Ultraviolet-B radiation effects on root growth traits, **(A)** longest root length, **(B)** total root length, **(C)** root surface area, and **(D)** root volume of 64 *indica* and *japonica* rice genotypes. Measurements were taken at 33 days after sowing and 21 days after UV-B treatment. The middle line indicates the median, and the box shows the range of the 25^th^ to 75^th^ percentiles of the total data. The whiskers indicate the interquartile range and the outer circle lines with high or low scores in each category.

**Figure 3** represents various root parameters for all *indica* and *japonica* rice lines. The highest decrease in root surface area was recorded for CL111 (238 cm^2^ plant^-1^, *japonica*), while a maximum increase was observed for the *japonica* genotype (RU1404156). Compared to control plants, the total root length decreased by around 31% for all, 28% for indica, and 34% for *japonica*, respectively (**Figure 3**). The total root length (TRL) and the longest root length (LRL) decreased maximum for a UV-B moderately tolerant genotype, NIPPONBARE. The highest values for TRL and LRL were recorded for IR09L179 (*indica*) and RU1304154 (*japonica*). The root surface area (RSA) values declined in response to UV-B for both *indica* and *japonica*, 32% and 35%, respectively (**Figure 3C**). Root volume (RV) decreased significantly for *japonica* (36%) and *indica* (34%) (**Figure 3D**).

**Figure 4:**
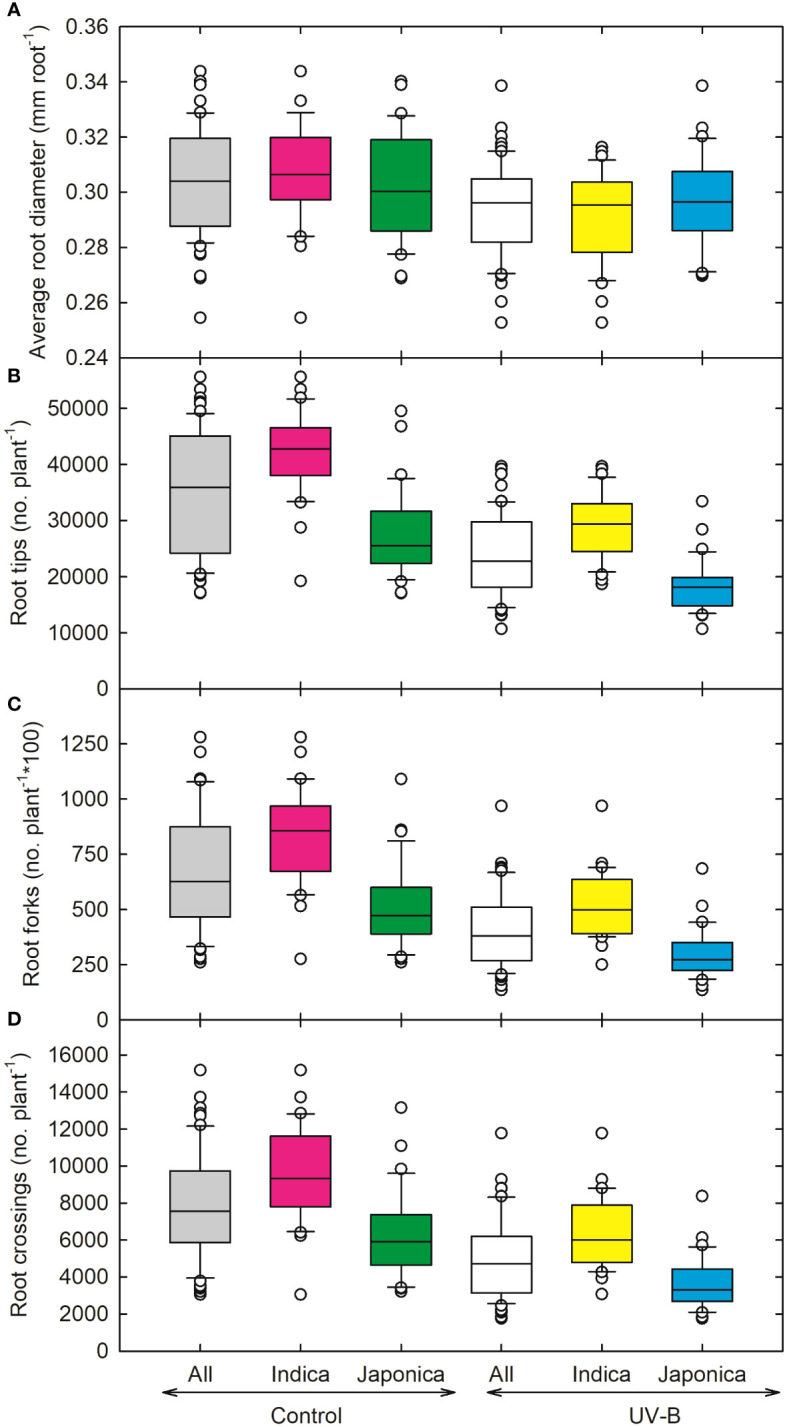
Ultraviolet-B radiation effects on root growth and developmental traits, **(A)** average root diameter, **(B)** root tips, **(C)** root forks, and **(D)** root crossings of 64 *indica* and *japonica* rice genotypes. Measurements were taken 33 days after sowing and 21 days after UV-B treatment. The middle line indicates the median, and the box shows the range of the 25^th^ to 75^th^ percentiles of the total data. The whiskers indicate the interquartile range and the outer circle lines with high or low scores in each category.

**Figure 5:**
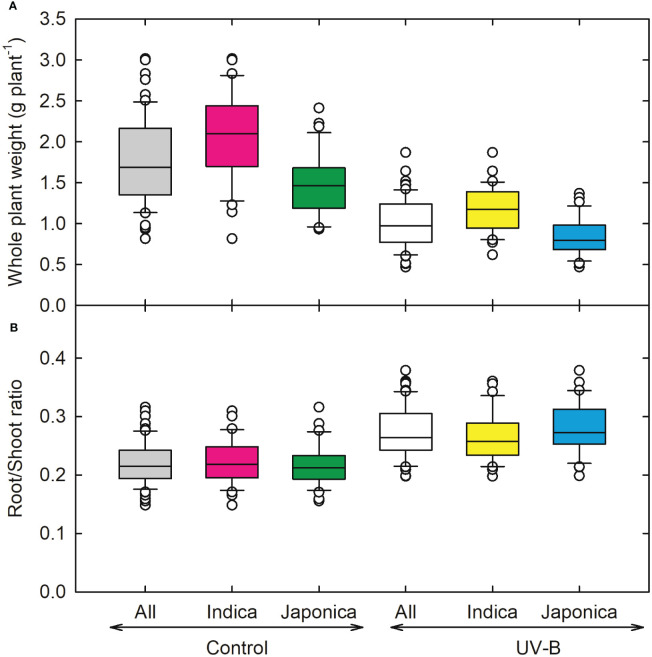
Ultraviolet-B radiation effects on growth traits, **(A)** whole plant weight, and **(B)** root and shoot ratio of 64 *indica* and *japonica* rice genotypes. Measurements were taken 33 days after sowing and 21 days after UV-B treatment. The middle line indicates the median, and the box shows the range of the 25^th^ to 75^th^ percentiles of the total data. The whiskers indicate the interquartile range and the outer circle lines with high or low scores in each category.

As compared to other root parameters, the average root diameter (RD) showed a lesser decrease (5%) for *indica* and (2%) *japonica* (**Figure 4A**), while recorded the highest value for RD for NIPPONBARE (0.32 cm root^-1^) and lowest for N-22 (*Aus*, 0.25 *cm* root^-1^). For root tips, *indica* genotype (CT18233-15-6-6-4-8-1) showed a maximum decrease (~21,939 no. plant^-1^), while recorded the highest values for *japonica* RU1404156 (23,137 no. plant^-1^). The relative decline in root tips for *indica* and *japonica* was around 32% and 33%, respectively. Maximum reduction in root fork and root crossing was observed for the UV-B *japonica* genotype (RU1504083), and RU1404156 (*japonica)* showed the highest value for both traits. The reduction in *indica* for root fork was around 37%, while for *japonica*, it was 42%. Root crossing decreased more for *japonica* (42%) as compared to *indica* (33%) genotypes (**Figures 4A–D**). Whole plant weight decreased maximum in an *indica* genotype (CT18244-9-4-4-2-1-2), followed by a *japonica* genotype (RU1204156). On average, 23% and 19% increases in root-to-shoot ratio were observed for *indica* and *japonica* (**Figures 5A, B**). The maximum root-to-shoot ratio was recorded in *japonica* Bowman, followed by the Cheniere genotype.

**Figure 6:**
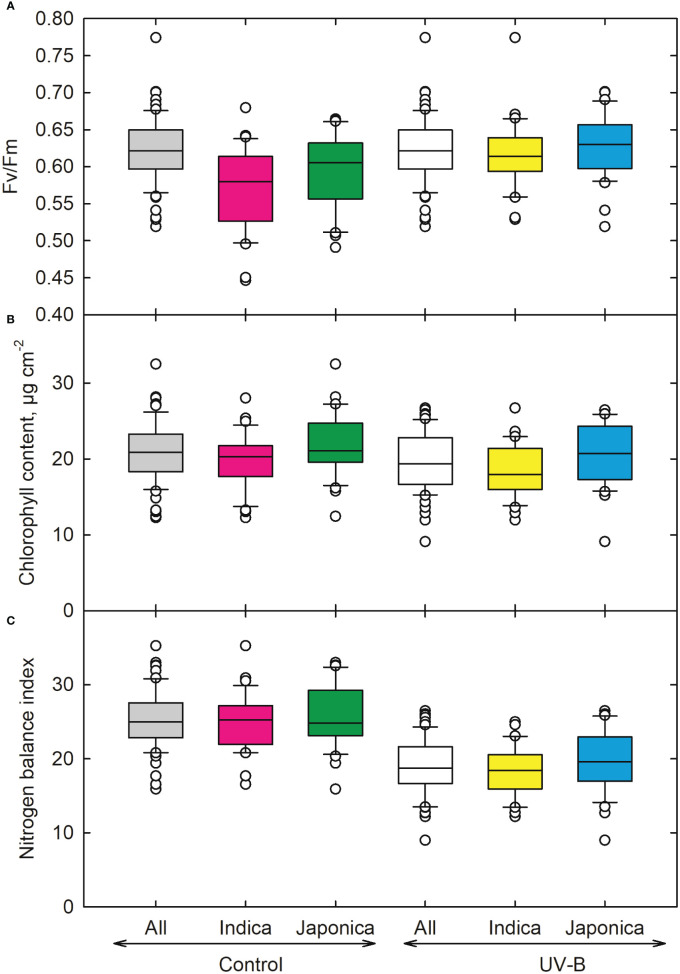
Ultraviolet-B radiation effects on **(A)** chlorophyll fluorescence, **(B)** chlorophyll index, and **(C)** nitrogen balance index of 64 *indica* and *japonica* rice genotypes. Measurements were made 33 days after sowing and 21 days after UV-B treatment. The middle line indicates the median, and the box shows the range of the 25^th^ to 75^th^ percentiles of the total data. The whiskers indicate the interquartile range, and the outer circle lines have high or low scores in each category.

The quantum efficiency of PSII (Fv/Fm) (**Figure 6A**) showed an 8% and 6% decrease in *indica* and *japonica*. The lowest value was recorded for the *indica* genotype (CT19561-3-57-2P-2-1-2-M) (**Table 1**). FEDEARROZ 21 (*indica)* (**Table 1**) showed the highest value for Fv/Fm. We also measured Chlorophyll (Chl) content for control and UV-B radiations for *indica* and *japonica* (**Figure 6B**). We observed a slight difference (7% for *indica* and 6% for *japonica*) for Chl content for both the sub-species (**Figure 6B**). An *indica* genotype (CT18247-12-8-1-4-2-2) showed maximum decrease followed by UV-B sensitive *japonica* genotype (CL111), respectively. We recorded the highest decrease in the nitrogen balance index for the *japonica* genotype CL111. Out of the two, *indica* showed a better nitrogen balance (26%) than *japonica* (24%) when exposed to UV-B radiation (**Figure 6C**).

**Table 1:** Classification of 64 *indica* and *japonica* rice genotypes into various ultraviolet (UV) B radiation tolerant groups based on cumulative UV-B response indices.

UV-B tolerance classification			
UV-B-sensitive	UV-B-moderately sensitive	UV-B-moderately tolerant	UV-B-tolerant
(14.16-15.99)	(16.00-17.81)	(17.82-19.64)	(>19.65)
CT18244-9-4-4-2-1-2 (14.16)	CT18372-8-1-6-3-1-5 (16.08)	CT6510-24-1-2 (18.00)	RU0603075 (19.48)
CL111 (14.84)	CT18233-15-6-6-4-8-1 (16.17)	CT19561-3-57-2P-2-1-2-M (18.18)	RU1504114 (19.58)
RU1204156 (15.02)	IrGA 409 (16.21)	CL151 (18.19)	IR04A115 (19.71)
RU1504122 (15.58)	N-22 (16.44)	Taggart (18.30)	RU1504197 (19.81)
El Paso 144 (15.72)	12DS-GMET-15 (16.54)	CT18237-13-11-3-3-5-1 (18.30)	Cheniere (19.88)
RU1504083 (15.76)	FEDEARROZ 2000 (16.55)	RoyJ (18.58)	IR65600-81-5-2-3 (20.00)
RU1404196 (15.94)	FEDEARROZ 21 (16.59)	IR86052-32-3-2 (18.62)	CT18593-1-7-2-2-5 (20.03)
	RU1204197 (16.70)	CT18615-1-5-1-2-1 (18.68)	WAB 56-125 (20.05)
	LAKAST (16.83)	CT6946-9-1-2-M-1P (18.77)	Thad (20.07)
	MILYANG 240 (16.99)	CT18245-4-7-1-1-2-1 (18.86)	Bowman (20.10)
	RU1504154 (17.08)	NIPPONBARE (18.97)	RU1504198 (20.20)
	CL163 (17.17)	HHZ 12-DT 10-SAL 1-DT 1 (19.13)	Sabine (20.24)
	Rex (17.22)	RU1303138 (19.19)	IR64-EMF NIL (20.27)
	CT18614-4-1-2-3-2 (17.26)	COLOMBIA XXI (19.41)	12DS-GMET-25 (20.29)
	FEDEARROZ MOCARE (17.29)	IR6 (19.46)	MERMENTAU (20.35)
	JES (17.40)	FEDEARROZ 473 (19.49)	IR09L179 (21.40)
	CT18247-12-8-1-4-2-2 (17.44)	RU1304154 (18.59)	CL271 (20.47)
	IR78049-25-2-2-2 (17.63)	RU1402174 (19.70)	Apo (20.70)
	INIA Tacuari (17.75)		HHZ 1-Y4-Y1 (21.11)
			RU1404156 (22.96)

The values in parenthesis indicate cumulative UV-B cumulative response indices calculated as described in the materials and methods section.

**Figure 7:**
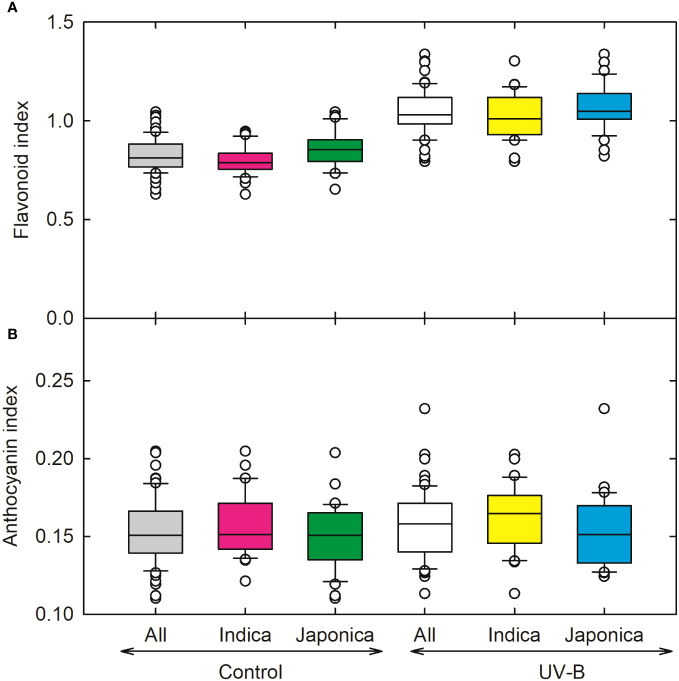
Ultraviolet-B radiation effects on **(A)** flavonoid index and **(B)** anthocyanin index of 64 *indica* and *japonica* rice genotypes. Measurements were made 33 days after sowing and 21 days after UV-B treatment. The middle line indicates the median, and the box shows the range of the 25^th^ to 75^th^ percentiles of the total data. The whiskers indicate the interquartile range and the outer circle lines with high or low scores in each category.

We recorded the highest value for the flavonoid index for *indica* genotype (CT18593-1-7-2-2-5), followed by the *japonica* genotype (CL111). Overall, in response to UV-B radiations, flavonoid content increased significantly, around (29%) in *indica* and (24%) in *japonica* (**Figure 7A**). Maximum values for anthocyanin were recorded for *indica* genotype (IR65600-81-5-2-3); overall changes in anthocyanin for *indica* and *japonica* were 4% and 2% under UV-B treatment (**Figure 7B**).

### Classification of UV-B tolerant and sensitive genotypes

**Figure 8:**
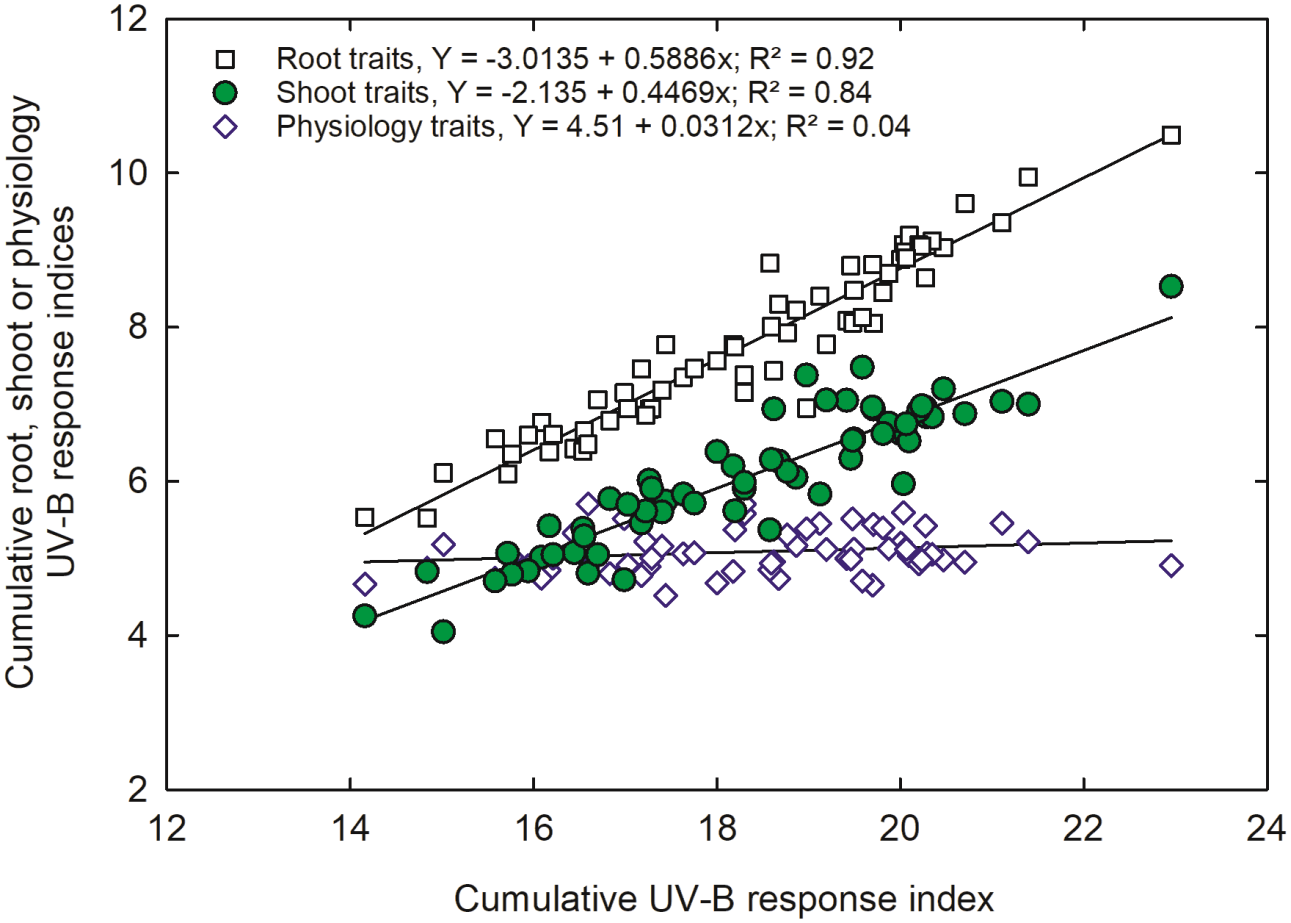
Correlation between combined cumulative shoot, root, or physiology UV-B-response index and total UV-B response index of all 64 *indica* and *japonica* rice genotypes traits.

A combined UV-B response index (CUV-BRI) was calculated to categorize the genotypes into UV-B sensitive, moderately sensitive, moderately tolerant, and tolerant. A strong correlation was observed for root and shoot traits (r^2^ = 0.92). In contrast, we observed a weaker correlation for physiology traits (**Figure 8**). The correlation indicated that root and shoot traits are more important and should be used as a criterion for genotype selection under UV-B conditions.

## Discussion

The results unveiled that UV-B has detrimental effects on rice growth and development. Dai et al. (1994) reported that rice’s sensitivity to UV-B’s plant height was much lower than leaf area and plant dry weight, which is corroborated by our study. Decreased plant height under UV-B could be due to one of the following reasons: (i) reduced carbohydrate content (Zhao et al., 2003), (ii) damaged cell components, and interaction of growth regulators (Ensminger and Schafer, 1992; Mohammed and Tarpley, 2013), (iii) shorter internodes rather than fewer nodes, (iv) decreased levels of growth hormone (indole acetic acid) in plants (Kakani et al., 2003b). A general growth delay has been documented to protect from UV-B radiation as it directly affects cell division (Kakani et al., 2003b).

In the present study, leaf area significantly decreased for both *indica* and *japonica* under UV-B. Reduction in leaf area due to UV-B is associated with damage to photosynthetic pigments. Thus, leaf area can be considered one of the critical parameters in selecting the rice genotype tolerant to UV-B. The decrease in light capture and photosynthetic activity due to UV-B was also supported by stomatal closure in plants (He et al., 2013; Martínez-Lüscher et al., 2013; Tossi et al., 2014). This stomata closure will decrease photosynthesis *in situ* and potentially decrease biomass production, as evident in our study (reduced plant, stem, and root weight). The decrease in chlorophyll pigments and photosynthesis reduced the plant height of most crop plants, including rice (Sah et al., 2022). Our study supports that the decreased stem and root weight in the present work could probably be through a reduction in cell division rather than reduced cell size, as reported in previous studies (Kakani et al., 2003b; Meng et al., 2019; Sah et al., 2022).

Roots are essential for growth and development, anchoring and structural support to plants for their growth substrate, facilitating water and nutrient uptake from the soil and sensing and responding to environmental signals such as biotic and abiotic stresses (Meng et al., 2019). In response to environmental stresses, root systems are continuously reshaped by the initiation and elongation of new roots throughout the growth period, facilitating the plant’s adaptation to biotic and abiotic stresses (Fukai and Cooper, 1995; Gowda et al., 2011; Meng et al., 2019). We observed a decrease in the root parameters as compared to the control. The probable reason could be the downregulation of photosynthesis and cell division in response to UV-B, which negatively affected rice genotypes’ root diameter and length (Meng et al., 2019; Yang et al., 2020; Sah et al., 2022).

Root length is an essential component of root architecture to survive in complex soil conditions. Root elongation is essentially driven by root apical meristems localized in the apical meristems of roots (Meng et al., 2019). We observed a slight negative impact on root length in response to UV-B supplementation. Root biomass decreased in response to UV-B. A potential reason for the decrease in root tips and the number of root crossings might be a modification in auxin transport within the roots under UV-B exposure (Yamamoto et al., 2007; Cho et al., 2014; Wang et al., 2014; Qin et al., 2017; Roychoudhry and Kepinski, 2022).

UV-B radiation directly impacts the DNA, proteins, and lipids (Sah et al., 2022). We observed a reduction in total chlorophyll content under UV-B. A previous study by Sah et al. (2022) also reported decreased chlorophyll a/b binding protein expression in some rice cultivars under UV-B. UV-B decreases the function of photosynthetic pigments, which can lead to a loss of photosynthetic capacity. Further, UV-B is also known to interfere with the light-harvesting complex (LHCII) of PSII and quantum efficiency in rice (Sah et al., 2022). In our study, a decline in quantum efficiency (Fv/Fm) was observed, which could present a reduction in the rate of primary charge separation and a decrease in the stabilization of charge separation and disconnection of some minor antennae from PSII (Mathur and Jajoo, 2015). The reduction in quantum efficiency of PSII can also be due to the downregulation of CP43 protein under UV-B (Sah et al., 2022). Decreased photosynthetic efficiency was also associated with reduced nitrogen balance, leading to lower leaf chlorophyll content.

Further, nitrogen is a fundamental constituent of many leaf cell components, particularly those associated with the photosynthetic apparatus, including carboxylation enzymes and chlorophyll. For these reasons, leaf nitrogen concentration strongly regulates maximum photosynthetic capacity (Rousseaux et al., 1998; Kakani et al., 2003b). Nitrogen balance decreased in both genotypes, indicating that UV-B has impacted the plant’s chlorophyll content, photosynthesis, and proteins. Hidema et al. (1996) also reported a decreased nitrogen content in rice plants, which is consistent with the present study.

Environmental conditions during early developmental stages appear to be essential to the development of plants as far as subsequent sensitivity to UV-B damage (Sullivan et al., 2007; Mathur et al., 2018). In response to the damaging effects of UV-B radiation, plants have developed some defense strategies to overcome or compensate for these detrimental effects. These defense mechanisms include an accumulation of UV-B absorbing pigments and UV-A photons to repair most UV-B-induced DNA damage (Britt, 1996). Carotenoids are yet other pigments that indirectly protect plants against the harmful impact of UV-B radiations by protecting photosynthetic apparatus.

Accumulating certain phenolic compounds such as anthocyanin and flavonoids plays a significant role in the UV-B repair mechanism in plants. Our results depict that flavonoid content increased in the *indica* rice genotype compared to *japonica*, indicating that the *indica* genotype may have a better UV-B repair mechanism. Markham et al. (1998) reported that in UV-tolerant rice cultivar M202, flavonoids play a more elusive role in the plant’s UV-B protection and screening. In plants, the synthesis of UV-absorbing flavonoids constitutes an effective non-enzymatic mechanism to mitigate photoinhibition and photooxidative damage caused by UV stress. This mechanism includes either reducing the penetration of incident UV radiation or acting as a quencher of reactive oxygen species (ROS). Increased flavonoid concentration under UV-B in the current study implies that plants maintain protective mechanisms to protect the underlying tissues against harmful radiation. The anthocyanin index did not show a significant change, but slight alterations in the anthocyanin index indicated that these are protective mechanisms in plants. Aggregating flavonoids, anthocyanin, and other UV-absorbing metabolites in tissues reduce epidermal UV transmittance and are crucial mechanisms in plant’s acclimation to changing UV environments.

## Conclusion

An extensive set of 64 rice genotypes, including *indica* and *japonica* sub-species, were screened and categorized as UV-B sensitive, moderately sensitive, moderately tolerant, and tolerant. UV-B had a detrimental effect on both rice ecotypes, but the damage differed for indica and japonica. Based on CUVBRI, we hypothesized that leaf area could be used as one of the early-stage indicators for UV-B stress tolerance in rice genotypes. The genotypes studied and identified will give breeders a more comprehensive range of genotype selection under UV-B treatments. The study can be further helpful in determining and selecting a better tolerant or susceptible rice line for better yield and production under abiotic stresses.

## Data availability statement

The original contributions presented in the study are included in the article/**Supplementary Material**. Further inquiries can be directed to the corresponding authors.

## Author contributions

SM: Writing – original draft, Writing – review & editing. RB: Writing – review & editing. SJ: Data curation, Methodology, Writing – review & editing. NK: Data curation, Writing – review & editing. VR: Funding acquisition, Writing – review & editing. WG: Writing – review & editing. KR: Conceptualization, Data curation, Software, Supervision, Visualization, Writing – review & editing.

## Supplementary material

Supplementary Table 2
**(A)** Descriptive statistics of physiological and dry weight traits of 64 rice genotypes under control (CNT) and ultraviolet (UV)-B. **P<* 0.05, ***P<* 0.01, ****P<* 0.001. **(B)** Descriptive statistics of physiological and dry weight traits of 64 rice genotypes under control (CNT) and ultraviolet (UV)-B. **P<* 0.05, ***P<* 0.01, ****P<* 0.001.
