# Supplementary material for: Impact of ultraviolet-B radiation on early-season morpho-physiological traits of indica and japonica rice genotypes
Source: Front Plant Sci. 2024 Mar 1;15:1369397. doi: 10.3389/fpls.2024.1369397 (PMC10941760; doi:10.3389/fpls.2024.1369397)
Supplement: Supplementary file 1 [file Table_1.docx]

**Supplementary material**

**Supplementary Table 1**

**Classification of 64 Rice Genotypes into *indica* and *japonica* Subtypes**

| **SLNO** | **Genotype** | **Type** |
| --- | --- | --- |
| 64 | N-22 | *(Aus)Australia* |
| 1 | 12DS-GMET-15 | *Indica* |
| 2 | 12DS-GMET-25 | *Indica* |
| 3 | Apo | *Indica* |
| 4 | COLOMBIA XXI | *Indica* |
| 5 | CT18233-15-6-6-4-8-1 | *Indica* |
| 6 | CT18237-13-11-3-3-5-1 | *Indica* |
| 7 | CT18244-9-4-4-2-1-2 | *Indica* |
| 8 | CT18245-4-7-1-1-2-1 | *Indica* |
| 9 | CT18247-12-8-1-4-2-2 | *Indica* |
| 10 | CT18372-8-1-6-3-1-5 | *Indica* |
| 11 | CT18593-1-7-2-2-5 | *Indica* |
| 12 | CT18614-4-1-2-3-2 | *Indica* |
| 13 | CT18615-1-5-1-2-1 | *Indica* |
| 14 | CT19561-3-57-2P-2-1-2-M | *Indica* |
| 15 | CT6510-24-1-2 | *Indica* |
| 16 | CT6946-9-1-2-M-1P | *Indica* |
| 17 | FEDEARROZ 2000 | *Indica* |
| 18 | FEDEARROZ 21 | *Indica* |
| 19 | FEDEARROZ 473 | *Indica* |
| 20 | FEDEARROZ MOCARE | *Indica* |
| 29 | HHZ 12-DT 10-SAL 1-DT 1 | *Indica* |
| 22 | HHZ 1-Y4-Y1 | *Indica* |
| 30 | IR04A115 | *Indica* |
| 23 | IR09L179 | *Indica* |
| 24 | IR6 (PAKISTAN) | *Indica* |
| 25 | IR64-EMF NIL | *Indica* |
| 26 | IR65600-81-5-2-3 | *Indica* |
| 27 | IR78049-25-2-2-2 | *Indica* |
| 28 | IR86052-32-3-2 | *Indica* |
| 31 | MILYANG 240 | *Indica* |
| 32 | WAB 56-125 | *Indica* |
| 44 | Bowman | *Japonica* |
| 33 | Cheniere | *Japonica* |
| 57 | CL111 | *Japonica* |
| 37 | CL151 | *Japonica* |
| 38 | CL163 | *Japonica* |
| 56 | CL271 | *Japonica* |
| 45 | El Paso 144 | *Japonica* |
| 50 | INIA Tacuari | *Japonica* |
| 49 | IrGA 409 | *Japonica* |
| 61 | JES | *Japonica* |
| 59 | LAKAST | *Japonica* |
| 53 | MERMENTAU | *Japonica* |
| 34 | Rex | *Japonica* |
| 52 | RoyJ | *Japonica* |
| 62 | RU0603075 | *Japonica* |
| 51 | RU1204156 | *Japonica* |
| 55 | RU1204197 | *Japonica* |
| 63 | RU1303138 | *Japonica* |
| 58 | RU1304154 | *Japonica* |
| 21 | RU1402174 | *Japonica* |
| 54 | RU1404156 | *Japonica* |
| 42 | RU1404196 | *Japonica* |
| 39 | RU1504083 | *Japonica* |
| 46 | RU1504114 | *Japonica* |
| 40 | RU1504122 | *Japonica* |
| 41 | RU1504154 | *Japonica* |
| 43 | RU1504197 | *Japonica* |
| 47 | RU1504198 | *Japonica* |
| 36 | Sabine | *Japonica* |
| 48 | Taggart | *Japonica* |
| 35 | Thad | *Japonica* |
| 60 | NIPPONBARE | *Japonica* |
