# Supplementary material for: Impact of ultraviolet-B radiation on early-season morpho-physiological traits of indica and japonica rice genotypes
Source: Front Plant Sci. 2024 Mar 1;15:1369397. doi: 10.3389/fpls.2024.1369397 (PMC10941760; doi:10.3389/fpls.2024.1369397)
Supplement: Supplementary Table 2 — (A) Descriptive statistics of physiological and dry weight traits of 64 rice genotypes under control (CNT) and ultraviolet (UV)-B. *P< 0.05, **P< 0.01, ***P< 0.001. (B) Descriptive statistics of physiological and dry weight traits of 64 rice genotypes under control (CNT) and ultraviolet (UV)-B. *P< 0.05, **P< 0.01, ***P< 0.001. [file Table_2.docx]

**Supplementary Material**

**Supplementary Table 2A**

|  | **PH (cm)** | **TN (no. plant^-1^)** | **LN**  **(no. main axis^-1^)** | **LA**  **(cm^2^plant^-1^)** | **RS** | **LRL (cm)** | **TRL**  **(cmplant^-1^)** | **RSA**  **(cm^2^plant^-1^)** | **RD**  **(mm root^-1^)** | **RV cm^3^plant^-1^)** | **Tips (no.plant^-1^)** | **Forks (no.plant^-1*^100)** | **Crossings (no. plant^-1^)** |
| --- | --- | --- | --- | --- | --- | --- | --- | --- | --- | --- | --- | --- | --- |
| **Control (CNT)** | | | | | | | | | | | | | |
| Minimum | 11.5 | 2.0 | 3.0 | 64.4 | 0.10 | 26.0 | 1729.1 | 153.0 | 0.2 | 1.1 | 8708.0 | 12468.0 | 1544.0 |
| Maximum | 29.0 | 9.0 | 7.0 | 473.0 | 0.52 | 66.0 | 9361.6 | 989.7 | 0.4 | 8.5 | 67934.0 | 156864.0 | 18606.0 |
| Mean | 17.6 | 4.8 | 3.7 | 212.3 | 0.22 | 44.9 | 5248.6 | 504.7 | 0.3 | 3.9 | 34876.7 | 67146.8 | 7876.6 |
| SD | 2.5 | 1.5 | 0.8 | 91.8 | 0.06 | 6.4 | 1661.1 | 174.4 | 0.0 | 1.5 | 12488.8 | 30687.6 | 3557.8 |
| *indica* | 17.4 | 5.3 | 3.9 | 265.7 | 0.22 | 44.3 | 5976.7 | 578.5 | 0.3 | 4.5 | 42216.4 | 82903.9 | 9438.0 |
| *japonica* | 17.6 | 4.3 | 3.5 | 154.6 | 0.22 | 45.4 | 4491.8 | 428.0 | 0.3 | 3.3 | 27419.1 | 50927.2 | 6259.2 |
| Ecotype difference | ns | ******* | ******* | ******* | ns | ns | ******* | ******* | ns | ******* | ******* | ******* | ******* |
| Genotype | ******* | ****** | ns | ******* | ***** | ns | ******* | ******* | ******* | ******* | ******* | ******* | ******* |
| **UV-B** | | | | | | | | | | | | | |
| Minimum | 7.5 | 1.0 | 3.0 | 11.5 | 0.07 | 21.0 | 509.4 | 45.0 | 0.2 | 0.3 | 4254.0 | 4105.0 | 479.0 |
| Maximum | 22.0 | 8.0 | 6.0 | 221.0 | 0.48 | 73.0 | 7926.7 | 708.9 | 0.4 | 5.7 | 48716.0 | 113482.0 | 16418.0 |
| Mean | 13.7 | 4.1 | 3.6 | 100.6 | 0.27 | 43.4 | 3603.1 | 335.3 | 0.3 | 2.5 | 23717.3 | 40720.1 | 4993.8 |
| SD | 2.3 | 1.4 | 0.6 | 44.3 | 0.08 | 7.7 | 1404.0 | 138.4 | 0.0 | 1.1 | 9179.9 | 22328.6 | 2817.6 |
| *indica* | 13.3 | 4.8 | 3.6 | 121.1 | 0.27 | 43.7 | 4250.8 | 394.7 | 0.3 | 2.9 | 28873.5 | 52260.6 | 6354.5 |
| *japonica* | 13.9 | 3.5 | 3.5 | 79.8 | 0.28 | 43.1 | 2958.6 | 277.9 | 0.3 | 2.1 | 18447.1 | 29362.2 | 3637.4 |
| Ecotype difference | * | *** | ns | *** | ns | ns | *** | *** | ns | *** | *** | *** | *** |
| Genotype | *** | *** | ns | *** | ns | * | *** | *** | ** | *** | *** | *** | *** |
| Genotype (G) | *** | *** | ns | *** | * | * | *** | *** | *** | *** | *** | *** | *** |
| Treatment (T) | *** | *** | * | *** | *** | * | *** | *** | *** | *** | *** | *** | *** |
| G x T | ** | ns | ns | *** | ns | ns | ns | ns | ns | ns | ns | ns | ns |
| **Percent change = (UV-B/CNT x 100)** | | | | | | | | | | | | | |
| Mean | -22.1 | -15.3 | -4.5 | -52.6 | 23.9 | -3.3 | -31.4 | -33.6 | -3.4 | -35.7 | -32.0 | -39.4 | -36.6 |
| *indica* | -23.6 | -10.7 | -7.9 | -54.4 | 19.3 | -1.3 | -28.9 | -31.8 | -4.5 | -34.5 | -31.6 | -37.0 | -32.7 |
| *japonica* | -21.1 | -20.0 | -1.8 | -48.4 | 29.0 | -5.1 | -34.1 | -35.1 | -1.7 | -36.0 | -32.7 | -42.3 | -41.9 |
| **Correlation between treatments** | | | | | | | | | | | | | |
| Correlation | 0.62 | 0.80 | 0.12 | 0.74 | 0.36 | 0.28 | 0.7 | 0.74 | 0.23 | 0.70 | 0.7 | 0.8 | 0.8 |
| Significance | *** | *** | ns | *** | ** | * | *** | *** | ns | *** | *** | *** | *** |

PH: Plant height, TN: Tiller number, LN: Leaves on the main axis, LA: Leaf area; RS: Root to shoot ratio, LRL: Longest root length; RSD: Root surface area, RD: Root diameter; RV: Root volume.

**Supplementary Table 2B**

|  | **LWT**  **(g plant^-1^)** | **StWT**  **(g plant^-1^)** | **RWT**  **(g plant^-1^)** | **SHWT**  **(g plant^-1^)** | **TDM**  **(g plant^-1^)** | **Chl** | **Flav** | **Anth** | **NBI** | **Fv/Fm** |
| --- | --- | --- | --- | --- | --- | --- | --- | --- | --- | --- |
|  |  |  |  |  |  |  |  |  |  |  |
|  |  |  |  |  |  |  |  |  |  |  |
| **Control (CNT)** | | | | | | | | | |  |
| Minimum | 0.17 | 0.10 | 0.07 | 0.27 | 0.35 | 6.8 | 0.55 | 0.09 | 9.6 | 0.31 |
| Maximum | 1.86 | 1.62 | 1.29 | 3.48 | 4.05 | 35.8 | 1.09 | 0.24 | 38.6 | 0.73 |
| Mean | 0.84 | 0.61 | 0.32 | 1.44 | 1.76 | 20.9 | 0.83 | 0.15 | 25.3 | 0.58 |
| SD | 0.30 | 0.26 | 0.15 | 0.55 | 0.68 | 5.0 | 0.11 | 0.03 | 5.4 | 0.09 |
| *indica* | 0.96 | 0.70 | 0.37 | 1.66 | 2.03 | 20.1 | 0.80 | 0.16 | 25.0 | 0.57 |
| *japonica* | 0.70 | 0.51 | 0.27 | 1.21 | 1.48 | 21.9 | 0.86 | 0.15 | 25.7 | 0.60 |
| Ecotype difference | ******* | ******* | ******* | ******* | ******* | ***** | ****** | ns | ns | ******* |
| Genotype | ******* | ******* | ******* | ******* | ******* | ******* | ******* | ******* | ******* | ns |
| **UV-B** | | | | | | | | | |  |
| Minimum | 0.04 | 0.02 | 0.01 | 0.06 | 0.07 | 1.6 | 0.58 | 0.10 | 1.9 | 0.41 |
| Maximum | 0.89 | 0.93 | 0.53 | 1.75 | 2.13 | 32.8 | 1.57 | 0.27 | 33.2 | 0.99 |
| Mean | 0.46 | 0.33 | 0.21 | 0.79 | 1.00 | 19.6 | 1.04 | 0.16 | 19.0 | 0.62 |
| SD | 0.18 | 0.17 | 0.10 | 0.34 | 0.43 | 5.3 | 0.17 | 0.03 | 5.2 | 0.07 |
| *indica* | 0.54 | 0.39 | 0.25 | 0.93 | 1.17 | 18.7 | 1.03 | 0.16 | 18.4 | 0.61 |
| *japonica* | 0.38 | 0.27 | 0.18 | 0.65 | 0.84 | 20.6 | 1.06 | 0.15 | 19.7 | 0.63 |
| Ecotype difference | *** | *** | *** | *** | *** | * | ns | ns | ns | *** |
| Genotype | *** | ** | ** | *** | *** | *** | * | *** | *** | ns |
| Genotype (G) | *** | *** | *** | *** | *** | *** | *** | *** | *** | *** |
| Treatment (T) | *** | *** | *** | *** | *** | *** | *** | ns | *** | *** |
| G x T | ns | ns | ns | ns | ns | ns | ns | ns | ns | ns |
| **Percent change = (UV-B/CNT x 100)** | | | | | | | | | |  |
| Mean | -44.7 | -46.5 | -32.9 | -45.5 | -43.2 | -6.4 | 26.3 | 2.8 | -25.0 | 6.8 |
| *indica* | -43.6 | -45.0 | -33.4 | -44.2 | -42.2 | -6.9 | 28.6 | 3.8 | -26.6 | 7.7 |
| *japonica* | -45.1 | -47.7 | -31.0 | -46.2 | -43.5 | -6.1 | 24.2 | 2.1 | -23.6 | 5.5 |
| **Correlation between treatments** | | | | | | | | | |  |
| Correlation | 0.72 | 0.46 | 0.58 | 0.61 | 0.61 | 0.58 | 0.54 | 0.59 | 0.44 | 0.36 |
| Significance | *** | *** | *** | *** | *** | *** | *** | *** | *** | ** |

LWT: Leaf dry weight; NBI: Nitrogen balance index; RWT: Root dry weight; SHWT: Shoot dry weight; StWT: Stem dry weight; TDM: Total dry mass.

Anth: Anthocyanin; Chl: Chlorophyll; Flav: Flavonoids; Fv/Fm: Quantum efficiency of Photosystem II.
